# Comprehensive Analysis of Differentially Expressed mRNA, lncRNA and circRNA and Their ceRNA Networks in the Longissimus Dorsi Muscle of Two Different Pig Breeds

**DOI:** 10.3390/ijms20051107

**Published:** 2019-03-04

**Authors:** Jing Wang, Qiaoling Ren, Liushuai Hua, Junfeng Chen, Jiaqing Zhang, Hongjie Bai, Haili Li, Bin Xu, Zhihai Shi, Hai Cao, Baosong Xing, Xianxiao Bai

**Affiliations:** 1 Henan Key Laboratory of Farm Animal Breeding and Nutritional Regulation, Institute of Animal Husbandry and Veterinary Science, Henan Academy of Agricultural Sciences, Zhengzhou 450002, China; wangjing_0407@163.com (J.W.); renql@163.com (Q.R.); hualiushuai@163.com (L.H.); afeng008@163.com (J.C.); jiaqingzhang1982@163.com (J.Z.); hongjiebai@163.com (H.B.); hailili_1981@163.com (H.L.); binxu1982@163.com (B.X.); zhihaishi88@163.com (Z.S.); 2 Henan Xing Rui Agriculture and Animal Husbandry Technology Co., LTD, Xinyang 465550, China; haicao6613@163.com

**Keywords:** ceRNA, lncRNA, circRNA, pig, skeletal muscle

## Abstract

Circular RNA (circRNA) and long non-coding RNA (lncRNA) are known to participate in adipogenesis and myogenic differentiation, but their impact on porcine muscle traits is not well understood. We compared their expressional profiles in the longissimus dorsi muscle of Chinese Huainan pigs (HN, the fat type) and Western commercial Duroc × (Landrace × Yorkshire) (DLY, the thin type) pigs, and 854 mRNAs, 233 lncRNAs, and 66 circRNAs (*p* < 0.05 and | log_2_FoldChange | >1) were found to be differentially expressed. The differentially expressed mRNA and circRNA parental genes were enriched in the Wnt signaling pathway (adipogenesis), the transition between fast and slow fibers (myogenic differentiation), and alanine, aspartate and glutamate metabolism (pork flavor). The potential lncRNAs/circRNAs-miRNAs-mRNAs regulatory networks shared *MYOD1*, *PPARD*, miR-423-5p and miR-874, which were associated with skeletal muscle muscular proliferation, differentiation/regeneration and adipogenesis. Taken together, these differentially expressed non-coding RNAs may be involved in the molecular basis of muscle traits, acting as the competing endogenous RNA (ceRNA) for miRNAs.

## 1. Introduction

In the pork industry, meat quality is an important economic trait, including the color, marbling, intramuscular fat (IMF) content, and firmness [1]. By its very nature, pork quality is associated with muscle growth and fatness, and it is a complex trait that is affected by the environment, feeding, management, slaughter conditions, and most importantly, genetics [2]. Compared with introduced pigs, the Chinese indigenous pigs show a redder pork color, higher IMF content, and thinner muscle fibers [3]. Huainan pigs, a typical indigenous Chinese fat-type pigs, are mainly distributed in Henan province and show a high litter size, roughage- and heat-resistance, and particularly, a high fat deposition [4]. By contrast, the Duroc× (Landrace × Yorkshire) (DLY) pig, which originates in the West and currently has the largest pork market share in China, shows a faster growth rate, higher meat productivity, higher lean meat percentage, and additional economic benefits [5]. These two breeds represent different muscle traits: the HN is a fat-type line, with a slower growth rate, while the DLY is a thin-type line, with a faster growth rate. Therefore, these two breeds are valuable for researching porcine muscle growth and fatness [3].

Advances in sequencing technology have revealed that the biological processes are regulated, not only by protein-coding RNA-(mRNA), but also by non-coding RNA (ncRNA), such as micro RNA (miRNA), long non-coding RNA (lncRNA), and circular RNA (circRNA). There have been several studies on how mRNA and miRNA affect the porcine muscle traits. For example, miR-23a was found to influence meat quality by reducing the composition of slow myosin heavy chain isoforms through myocyte enhancer factor 2C (*MEF2C*) [6]. The miR-29a regulated the level of type III collagen by reducing collagen type III alpha 1 (*COL3A1*) [7]. In intramuscular preadipocyte, the overexpression of miR-17-5p inhibited nuclear receptor co-activator 3 (*NCOA3*), fatty acid binding protein 4 (*FABP4*) and peroxisome proliferator-activated receptor gamma (*PPARG*) expressions and reduced preadipocyte differentiation [8]. In porcine intramuscular preadipocyte, miR-34a and forkhead box O1 (*FoxO1*) regulated platelet-derived growth factor receptor alpha (*PDGFRα*) and promoted IMF deposition through the Erk signaling pathway [9]. It was reported that miR-499 and miR-22 were potential candidates, associated with the drip loss of pork [10]. The overexpression of miR-125a-5p inhibited the differentiation of porcine intramuscular preadipocyte and reduced the total saturated fatty acids (SFA) content and monounsaturated fatty acids (MUFA)/SFA ratios, so miR-125a-5p may be a novel regulator of porcine IMF [11]. There also were reports on the effect of lncRNAs on porcine meat quality. For example, the lncRNAs of intramuscular adipocytes of Bamei pigs (fat type) were compared with those of Large White pigs (lean type), and it was found that lnc_000414 was closely related to fat synthesis [12]. Long non-coding RNA maternally expressed gene 3 (lncRNA MEG3) participated in the regulation of skeletal muscle development, and it can be used as a candidate gene for improving meat production traits in pigs [13]. SYISL (SYNPO2 intron sense-overlapping lncRNA) promoted myoblast proliferation and fusion but inhibited myogenic differentiation by interacting with polycomb repressive complex 2 (*PRC2*) [14]. LncRNAs-AK143003, which was significantly regulated by myogenic differentiation 1 (*MYOD1*), reduced the accumulation of myogenic marker genes and played a role in controlling muscle differentiation [15]. By formatting a sense–antisense RNA duplex with *PU.1* (also known as SPI1, spleen focus forming virus (*SFFV*) proviral integration oncogene spi1) mRNA, PU.1 AS lncRNA (vs. its mRNA translation) promoted adipogenesis [16].

Recently, there were several researches on the effect of circRNAs on the proliferation, differentiation, and migration of myoblast. For example, in rat, rno-circ_005717 (circDiaph3) promoted vascular smooth muscle cells (VSMCs) differentiation by up-regulated diaphanous-related formin-3 (DIAPH3) and supported its proliferation and migration by up-regulated insulin, such as the growth factor 1 receptor (*IGF1R*) [17]. In mouse myoblast cell line-C2C12, circZfp609 (circular RNA, derived from Zinc Finger Protein 609) could repress the myogenic differentiation by sponge miR-194-5p to sequester its inhibition on the apoptosis regulator (BCL2) associated with transcription factor 1 (*BCLAF1*) [18]. In chicken, circSVIL (circRNA generated from the exon 6 to 14 of the supervillin (SVIL) gene) promoted embryonic skeletal muscle development by upregulating the level of c-Jun Proto-Oncogene (*c-JUN*) and *MEF2C* via miR-203 [19]. In cattle, the circFGFR4 (circRNA generated from the fibroblast growth factor receptor 4 (FGFR4) gene)-miR-107-Wnt3a axis promoted bovine primary myoblasts differentiation [20]. From these findings, it can be inferred that circRNAs play critical roles in muscle development. Meanwhile, there were several researches that reported that circRNAs were associated with adipogenesis and lipid metabolism, and it was reported that, in the subcutaneous preadipocytes of Chinese Erhualian pigs, 902, 787, 710, 932, and 850 DE circRNAs were identified between D2 and D0, between D4 and D2, between D8 and D4, between D4 and D0, and between D8 and D0, respectively [21]. In the subcutaneous adipose tissue of Large White pigs and Laiwu pigs, 275 differentially expressed circRNAs were found, and the target genes of circRNA_26852 and circRNA_11897 were associated with adipocyte differentiation and lipid metabolism [22]. The circRNA_021412/miR-1972/LPIN1 (*lipin 1*) axis participated in the regulation of hepatic steatosis [23]. In a gastric cancer patient, exosome-delivered circRNA (ciRS-133) was involved in WAT browning [24]. From these findings, it can be inferred that circRNAs participated in the regulation of muscle development and lipid metabolism, but their effects on porcine muscle traits remains unclear. Thus, we first compared the expressional level of mRNAs, lncRNAs, and circRNAs in the longissimus dorsi (LD) muscle of HN and DLY. Then, we constructed and preliminarily verified the ncRNA-miRNA-mRNA regulatory networks to identify the key factors involved in porcine muscle traits.

## 2. Results

### 2.1. Predictions and Properties of lncRNAs and circRNAs in Porcine LD Muscle

**Figure 1:**
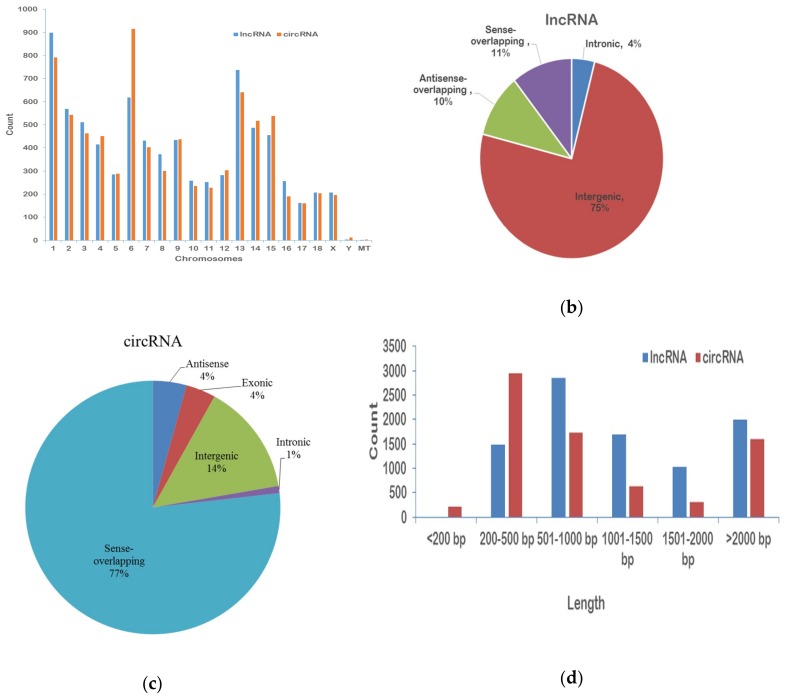
Comparison of the features of lncRNA and circRNAs in the porcine longissimus muscle. Chromosomes (**a**), genome (**b**,**c**) and length distribution (**d**) of lncRNA and circRNAs. Note: MT shows mitochondria.

After the redundant and low-quality reads were removed, 83.61 and 83.58 million clean reads were obtained from the LD muscles of HN and DLY pigs, respectively. The datasets were submitted to the National Center for Biotechnology Information Gene Expression Omnibus, with accession number GSE86919. In total, 9068 lncRNAs and 6988 circRNAs were identified in all chromosomes. Additionally, there were four circRNAs in mitochondrial DNA (mtDNA) (Figure 1a), including intergenic, intronic, antisense- and sense-overlapping lncRNAs/circRNAs, and in addition, exonic circRNAs. The majority (53.6%) of the lncRNAs were intergenic, and the minority (4%) were intronic. However, the majority (77%) of circRNAs were sense-overlapping, followed by intergenic (14%) circRNAs, and only a small quantity (1%) were located in introns (Figure 1b,c). The lengths of lncRNAs ranged from 203 to 21,809 bp, and for circRNAs, they were 110 to 99,406 bp. The majority (66.50%) of the lncRNAs were less than 1500 bp, and approximately half of the identified circRNAs (42.5%) were less than 500 bp (Figure 1c), which coincides with the features of lncRNAs and circRNAs [25,26]. 

### 2.2. Expressional Difference of mRNA, lncRNAs and circRNAs between HN and DLY Pigs

**Figure 2:**
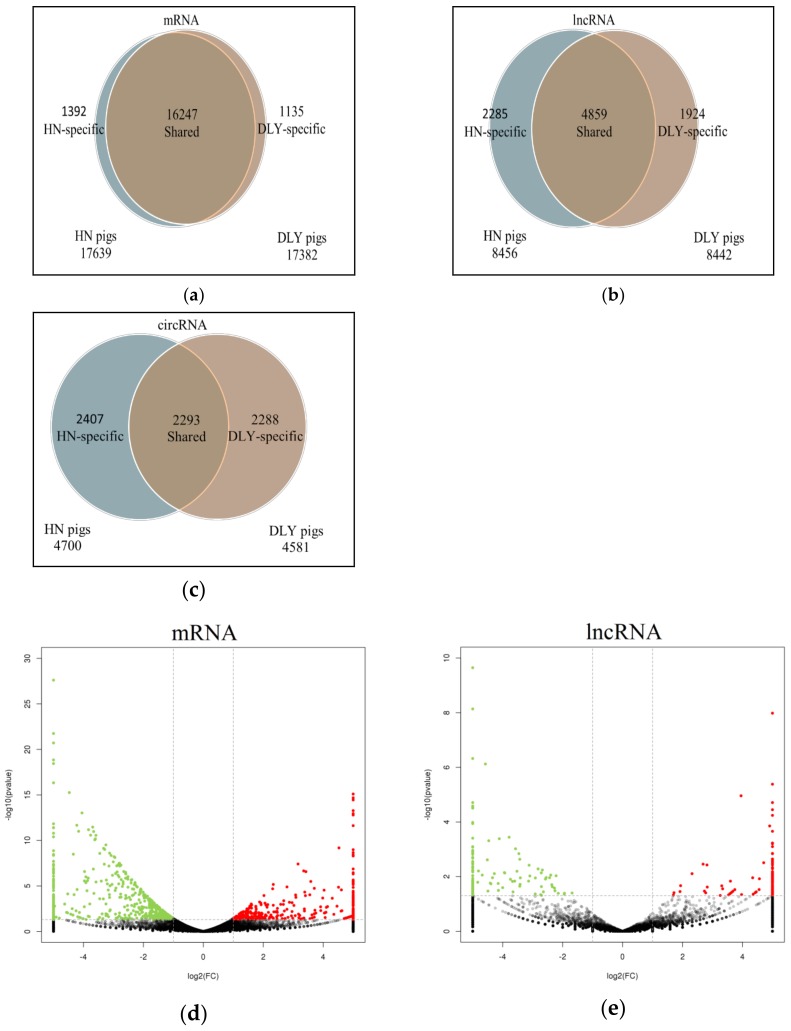

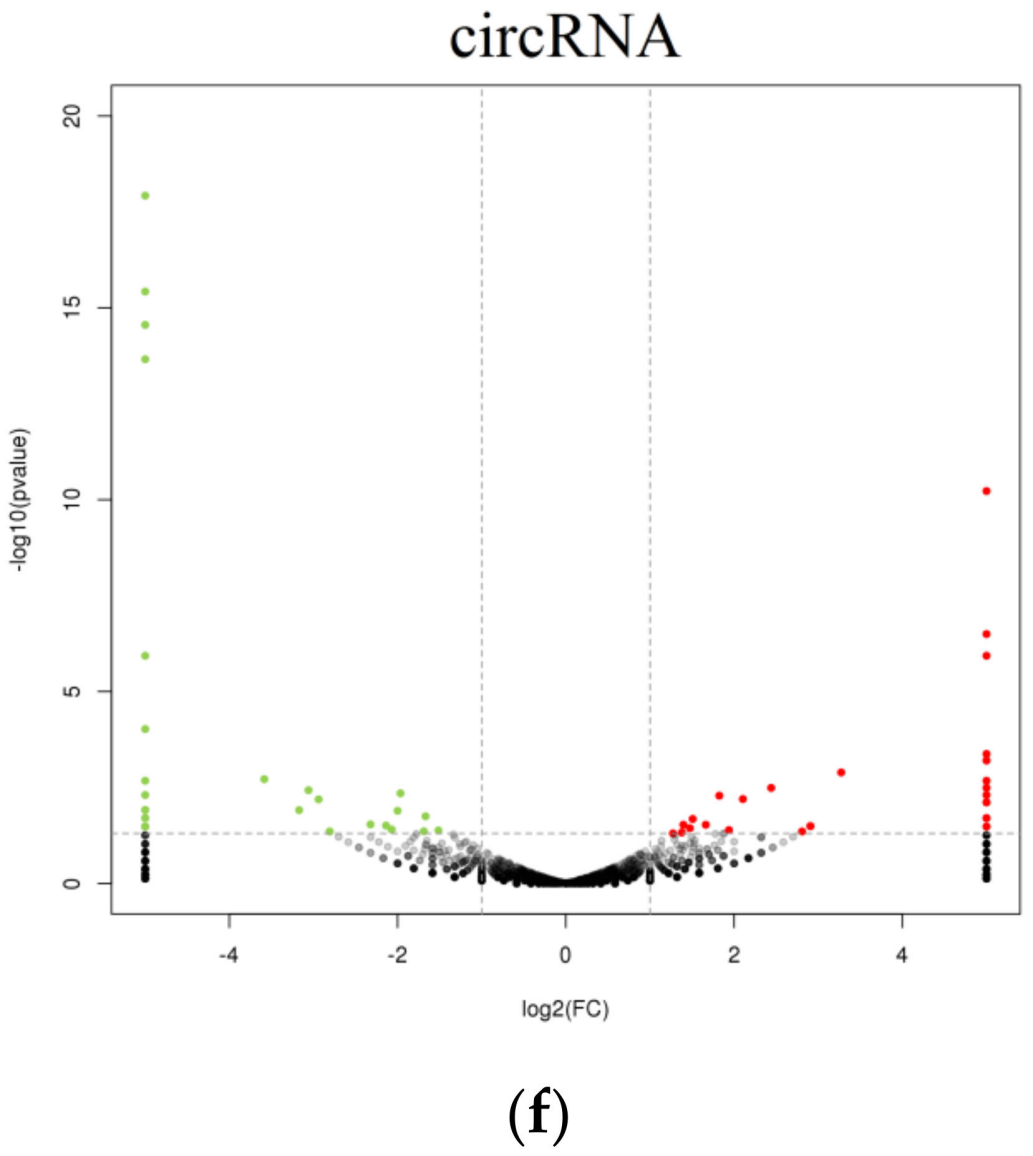
Comparative analysis of mRNAs, lncRNAs, and circRNAs between Huainan (HN) and Duroc × (Landrace × Yorkshire) (DLY) pigs. (**a**) The specific mRNAs (a), lncRNAs (**b**), and circRNAs (**c**) shared between HN and DLY pigs. (b) The mRNAs (**d**), lncRNAs (**e**), and circRNAs (**f**) differentially expressed in HN and DLY pigs. Note: The red points showed the up- regulated mRNA, lncRNA, circRNAs, the green points showed the down-regulated mRNA, lncRNA, circRNAs, and the black points showed the equally expressed mRNA, lncRNA, circRNAs. The vertical dotted lines indicate | log_2_FC | = 1, and the horizontal dotted lines indicate *p* value = 0.05.

We found 16,247 (86.5%) mRNAs, 4,859 (53.6%) lncRNAs, and 2,293 (32.8%) circRNAs that were shared by both HN and DLY pigs (Figure 2a). The average FPKM/RPM values of these mRNAs, lncRNAs and circRNAs were 1074, 487, and 21, respectively. Compared with the DLY pigs, there were 854 mRNAs (including 320 up- and 534 down-regulated), 233 lncRNAs (including 110 up- and 123 down-regulated), and 66 circRNAs (including 32 up- and 34 down-regulated) that were differentially expressed in the HN pigs (Figure 2b and Tables S1–S3). The DELs and DECs were located in all chromosomes, except the Y chromosome and mtDNA.

### 2.3. Functional Analysis of DEMs and DECs

**Figure 3:**
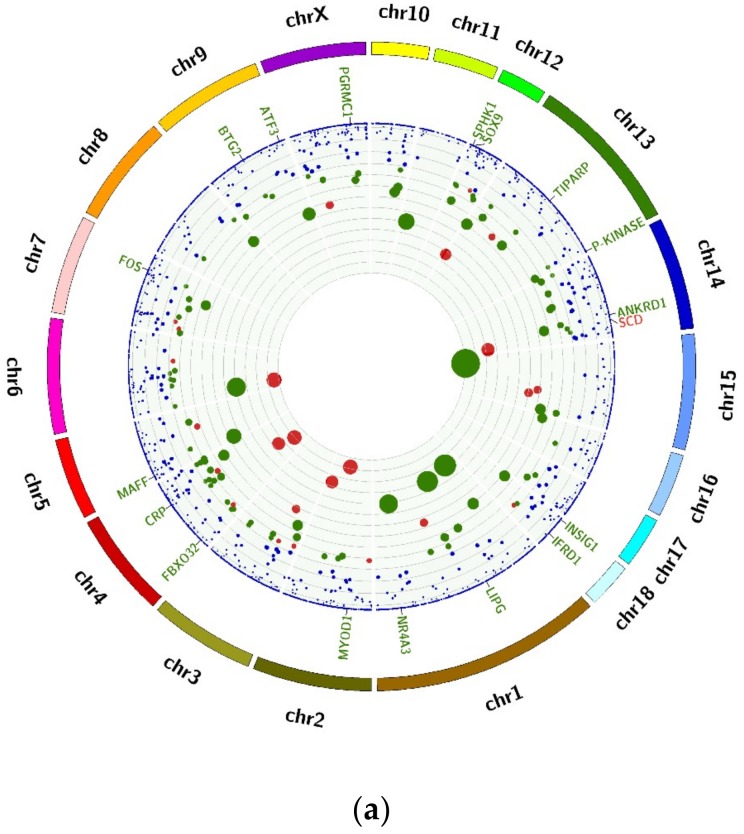

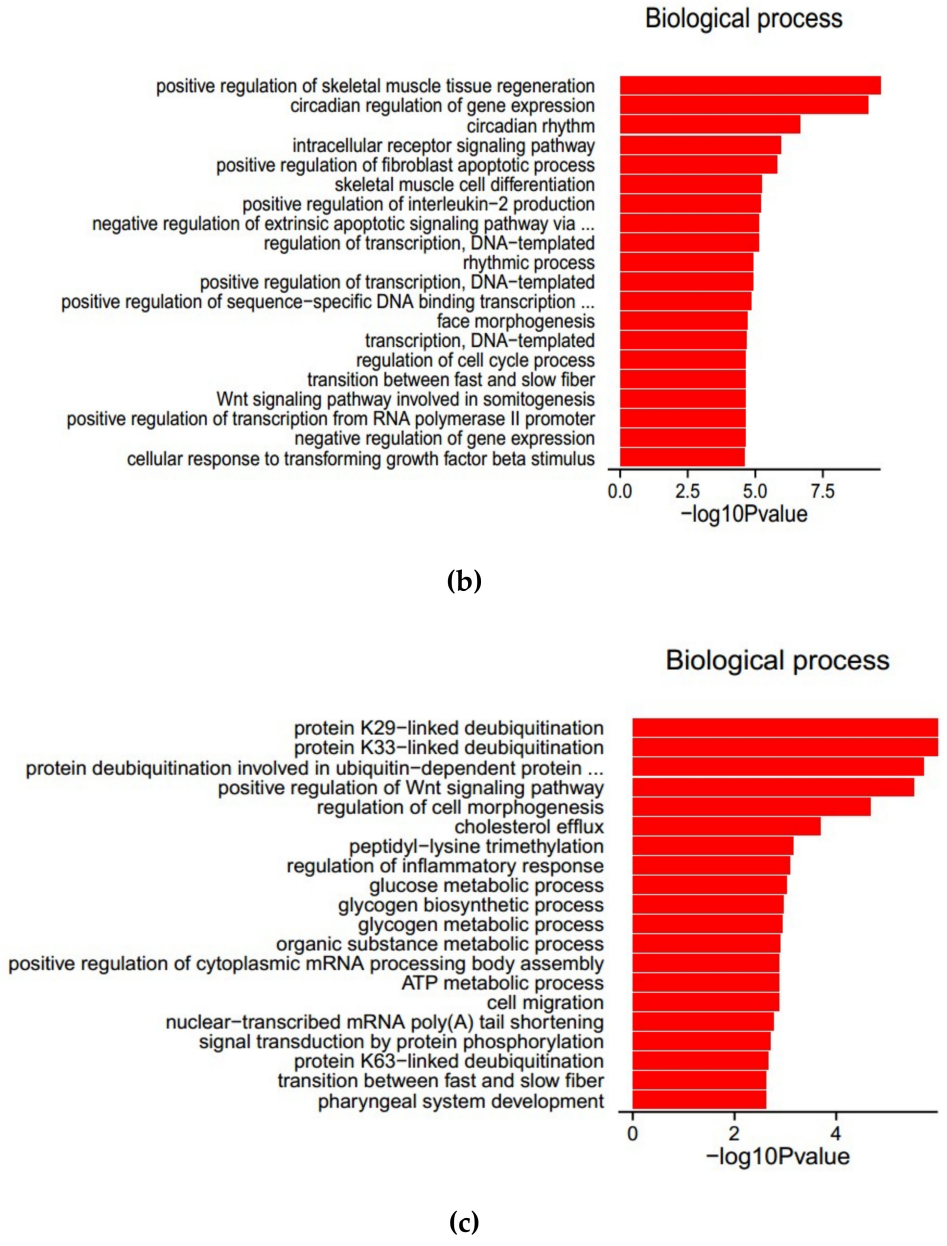
Functional analysis of genes (DEMs) and circRNAs (DECs) differentially expressed in Huainan (HN) and Duroc × (Landrace × Yorkshire) (DLY) pigs. (**a**). DEMs associated with muscle development and fatness (the outer ring represents 1 to 18 porcine autosomal and X, Y sex-chromosomes. The middle ring indicates the DEMs involved in muscle growth and development. The scatter plot in the inner ring represents genes present in the porcine muscle; the size of the solid circle stands for -log_10_(*p* value); the red symbols represent genes that were up-regulated in HN pigs; the green represents down-regulated genes; and the blue represents equally expressed genes); (**b**). Enriched biological process of DEMs; (**c**). Enriched biological process of DECs.

The 18 DEMs associated with muscle growth and fatness are shown in Figure 3a. The enriched GO terms for the DEMs included skeletal muscle tissue regeneration, skeletal muscle cell differentiation, translation between fast and slow fibers and the Wnt signaling pathway (Figure 3b). The DECs’ parent genes were associated with the Wnt signaling pathway and the transition between fast and slow fibers (Figure 3c). 

### 2.4. Construction of a Potential lncRNA/circRNA-miRNA-mRNA Regulatory Network

**Figure 4:**
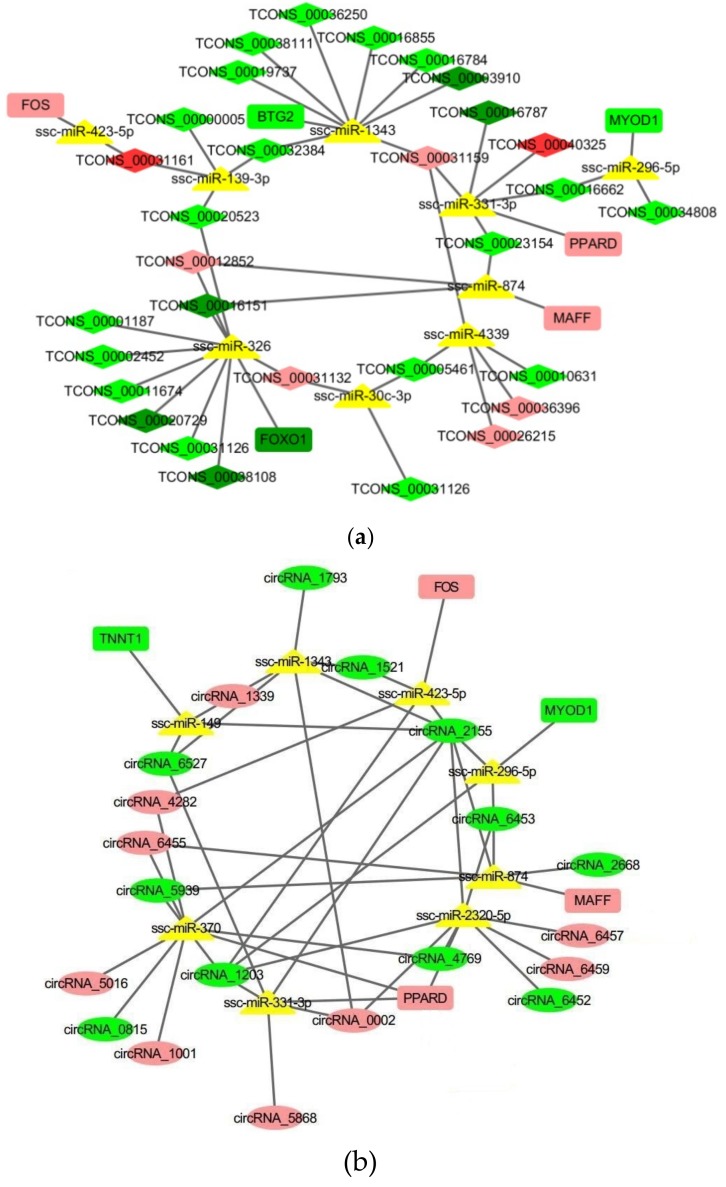
Network analysis of lncRNA-miRNA-mRNA (**a**) and circRNA-miRNA-mRNA (**b**). Square nodes represent mRNAs; triangular nodes represent miRNAs; diamond nodes represent lncRNAs; and circular nodes represent circRNAs. Red nodes represent the up-regulated, and the green ones represent the down-regulated transcripts, in Huainan (HN) and Duroc × (Landrace × Yorkshire) (DLY) pigs (a deeper color represents a higher difference in the expressional level between different breeds), and the yellow nodes represent the undetected transcripts.

As shown in Figure 4a, in the lncRNA-miRNA-mRNA network, there were 45 nodes and 47 connections between six mRNAs, nine miRNAs, and 30 lncRNAs. Meanwhile, the circRNA-miRNA-mRNA network (Figure 4b) included 33 nodes and 50 connections between five mRNAs, eight miRNAs and 20 circRNAs. There were several nodes were shared by these two networks, such as *MYOD1*, Peroxisome Proliferator Activated Receptor Delta (*PPARD*), miR-423-5p and miR-874.

### 2.5. Validation of the Expressional Level of the MYOD1-related Competitive Endogenous RNA (ceRNA)

**Figure 5:**
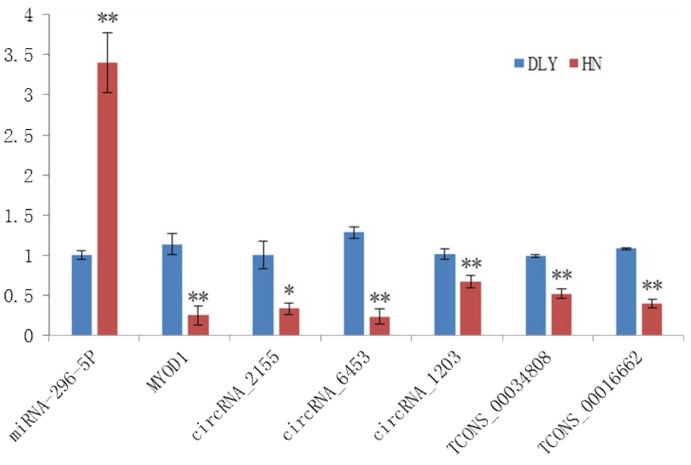
Validation of the expression of the *MYOD1*-related ceRNA network (miRNA-296-5p, circRNA_2155, circRNA_6453, circRNA_1203, TCONS_00034808, and TCONS_00016662) by qRT-PCR. Note: The *GAPDH* and *U6* were used as reference genes, the data represented the Mean±SD from 3 biological replicates, and each measurement was repeated 3 times. The fold change from the qPCR was calculated using the 2^-△△Ct^ method. ** indicates *p* < 0.01, * indicates *p* < 0.05.

To validate the potential interactions in the network, the expressional level of *MYOD1*-related ceRNAs were measured by RT-qPCR (Figure 5). The expressional level of miRNA-296-5p was up-regulated in the HN breeds. In contrast, its target genes, including *MYOD1*, circRNA_2155, circRNA_6453, circRNA_1203, TCONS_00034808, and TCONS_00016662, were down-regulated.

## 3. Discussion

Pork quality is a key economic trait. Thus, its molecular mechanism is worth researching. We compared, for the first time, the difference in the expressional profiles of mRNAs, lncRNAs, and circRNAs in the LD muscle of two pig breeds with different muscle types to identify the key factors involved in porcine muscle growth and fatness. Additionally, we constructed and briefly validated a potential ncRNA-miRNA-mRNA regulatory network, which provides new insight into the molecular mechanism of pork quality.

In the GO analysis of DEMs, the genes associated with muscle traits were visible in Circos plots in Figure 3a, 18 of which were significantly differentially expressed in these two breeds, and only *SCD* (stearoyl-coA desaturase) was up-regulated in the HN breed. These 18 genes included the genes that participated in muscle growth and development, such as *MYOD1*, *FOS* (Finkel-Biskis-Jinkins murine osteosarcoma viral oncogene homolog), and *IFRD1* (interferon related developmental regulator 1), and the genes associated with adipogenesis and lipid metabolism, such as *SCD*, *INSIG1* (insulin induced gene 1) and *LIPG* (lipase G, endothelial type). Meanwhile, in the GO analyses, the transition between fast and slow fibers for both DEMs and DECs was enriched. It is well known that the muscle fiber types have an influence on meat quality [27], for example, by reducing type IIB/X fibers, the cross-sectional area of fibers leads to an improvement of the meat quality [28]. Another shared enriched term was the Wnt signaling pathway, which is a vital pathway in adipogenesis [29]. These results indicated that the differences in muscle traits between these two breeds were not only associated with muscle development, but also with lipid deposition, and these differences were regulated not only by mRNAs, but also by circRNAs. 

While alanine, aspartate and glutamate were the non-essential amino acids, they played key roles in the pork taste and flavor [30]. In the current KEGG analysis, alanine, aspartate and glutamate metabolism pathways were enriched for both DEMs and DECs, for example, the D-aspartate oxidase (*DDO*) gene and circRNA_4504 from glutamine-fructose-6-phosphate transaminase 1 (*GFPT1*) were significantly up-regulated in HN pigs, which was consistent with the better taste of the native Chinese pigs, compared with the Western pigs [3,31]. 

LncRNAs participated in the regulation of different biological processes in different ways [32,33]. First of all, they could change the chromatin spatial conformation by directly binding to the target genes. As a result, the target genes’ expressional levels were changed. lncRNA Panct1 (an X-chromosome-associated intronic lncRNA) maintained mouse embryonic stem cell identity by the transient recruitment of transient octamer binding factor 1 (*TOBF1*) to genomic sites resembling the canonical Oct-Sox motif [34]. Secondly, lncRNAs regulated the target genes’ expression by interacting with the key transcription factors. For example, lncRNA plnc1 (which is transcribed from the upstream of the PPARG2) promoted adipogenic differentiation by reducing the methylation level of the CpG region in the *PPARG2* promoter and increasing its transcription [35]. Brown fat lncRNA (Blnc1) promoted brown adipocyte differentiation by forming a ribonucleoprotein complex with EBF transcription factor 2 (*EBF2*) to stimulate the thermogenic gene program [36]. Thirdly, the antisense lncRNAs could regulate their own mRNAs’ expression by directly binding with it, adiponectin AS lncRNA inhibited adipogenesis by attenuating adiponectin mRNA translation [37]. Recently, with the progress in the function of miRNAs, a new theory was proposed—the competing endogenous RNAs (ceRNA). Studies showed that mRNAs, pseudogene, lncRNAs and circRNAs regulated each other’s (with the same miRNA binding sites) expression by functioning as competing endogenous RNAs (miRNA sponge). For example, adipocyte differentiation-associated long noncoding RNA (lncRNA ADNCR) suppresses adipocyte differentiation by functioning as a ceRNA for miR-204 [38]. The Lnc-smad7 (terminal differentiation-induced ncRNA)/miR-125b/*smad7* (SMAD Family Member 7) and *IGF2* (insulin-like growth factor 2) axes promote myoblast differentiation and muscle regeneration [39]. CircRNA_010567 promotes myocardial fibrosis by functioning as a miR-141 sponge, which targets transforming growth factor beta 1 (*TGF-β1*) [40]. In this study, we found 69 nodes and 97 connections in the constructed ncRNA-miRNA-mRNA networks (Figure 4). Peroxisome proliferator-activated receptor delta (*PPARD*) was shared by these two networks and was the target gene of three miRNAs, and these miRNAs targeted five lncRNAs and 16 circRNAs. Another shared gene was *MYOD1*, and two lncRNAs and three circRNAs act as its ceRNA via miR-296-5p. As reported, *PPARD* was the vital regulator in adipogenesis [41] and lipid metabolism [42], and *MYOD1* was an important transcription factor during muscle development, so these ncRNAs may also participate in these processes by indirectly regulating the expressional level of *PPARD* and *MYOD1*. MiR-423-5p and miR-874 were shared by these two networks, and miR-423-5p targeted one lncRNA, four circRNAs and *FOS*. It was reported that miR-423-5p was involved in skeletal muscle development/regeneration [43], and *FOS* was associated with porcine skeletal muscle fiber traits [44]. Similarly, the miR-874, which was involved in the *PPAR* signaling pathway and affected bovine fat deposition [45], targeted three lncRNAs and five circRNAs. From these data, it is inferred that the identified DELs and DECs not only participate in muscle cell proliferation and differentiation, but also in adipogenesis by acting as ceRNAs. 

As for the economic considerations, an equal amount of the RNA sample from both the HN pigs and DLY pigs was pooled for sequencing. A similar approach was previously used in RNA sequencing [46,47,48,49]. Moreover, we compared our results with published articles, and the differentially expressed genes were also associated with adipogenesis and myogenic differentiation [50,51]. The expression trend of the *MYOD1*-related ceRNA network were preliminarily confirmed by qRT-PCR, and the results were consistent with RNA sequencing, but their functions and interactions needed further validation. RNA pulldown (chromatin conformation, pre-transcriptional level), ChIRP (interactions with RNA and protein, transcriptional level), and dual luciferase reporter systems (ceRNA mechanism, post-transcriptional level) can be used to validate these DELs’ and DECs’ functions in relation to porcine meat quality.

## 4. Materials and Methods

### 4.1. Ethics Approval

The experimental methods were performed in accordance with the guidelines of the Good Experimental Practices, adopted by the Institute of Animal Science. Additionally, all experimental protocols were approved by the Institute of Animal Science of the Henan Academy of Agricultural Sciences (code 2 May 2015).

### 4.2. Experimental Animals and Sample Collection

Six male HN and DLY pigs (three randomly-selected full-sib pigs from each breed, all with a similar birth weight) were obtained from the Henan Xing Rui Agricultural and Animal Husbandry Technology Co., LTD in Henan province, China. These pigs were castrated under anesthesia (surgery was performed under sodium pentobarbital anesthesia, and all efforts were made to minimize suffering), and they were housed under standard environmental conditions (including unregulated room temperatures and natural light) and were fed a standard diet three times a day and watered ad libitum [52]. All pigs were slaughtered when their body weight reached around 130 kg (after about 300 days for HN and 210 days for DLY). After slaughter, the longissimus dorsi (LD) muscle at the 6–7th rib of the right side of the carcass was obtained, immediately frozen in liquid nitrogen and stored at −80 °C until analysis.

### 4.3. Total RNA Isolation and Illumina Sequencing

The total RNA was isolated from the porcine LD muscle using TRIzol reagent (Invitrogen^™^, Carlsbad, CA, USA), according to the manufacturer’s instructions. DNase I (TaKaRa, Dalian, China) was used to remove the residual genomic DNA from the total RNA. Agarose gel electrophoresis, a Nano-Drop ND-2000 spectrophotometer (NanoDrop Products, Wilmington, DE, USA), and an Agilent 2100 Bioanalyzer (Agilent Technologies, Massy, France) were used to assess the integrity, purity, and quality of the isolated RNA. Samples with an RNA integrity number (RIN) value larger than eight were used for further analysis. For each breed, equal quantities of RNA, isolated from each individual, were pooled.

Ribosomal RNA (rRNA) was removed from the DNA-free RNA using the Ribo-ZeroTM kit (Epicentre, Madison, WI, USA). Next, the DNA and rRNA-free RNA were used to create a library using the NEBNext Ultra Directional RNA Library Prep Kit for Illumina (New England BioLabs Inc., Beverly, MA, USA). The library was sequenced with a HiSeq 2500 instrument (125 bp PE, Illumina, San Diego, CA, USA).

### 4.4. Read Mapping and Transcript Assembly

Clean data were obtained by removing the poor-quality bases and adapter sequences from the raw data using FASTQC (http://www.bioinformatics.babraham.ac.uk/projects/fastqc/) and the NGS QC TOOLKIT (http://59.163.192.90:8080/ngsqctoolkit/) [53]. TopHat2 software [54] was used to map the clean data to the porcine reference genome (Sscrofa10.2, ftp://ftp.ncbi.nlm.nih.gov/genomes/Sus_scrofa/). Cufflinks was used to perform the transcript assembly and abundance estimation [55].

### 4.5. Identification of lncRNA

The pipelines for lncRNAs identification were as follows: (1) Transcripts that were merged or were similar to the known porcine mRNAs and other small RNAs (rRNA, snRNA, snoRNAs, tRNAs, pre-miRNA, pseudogenes) were removed using cuffcompare software [56]. (2) The transcripts, annotated as “i (a transfrag falling entirely within a reference intron)”, “u (unknown, intergenic transcript)”, “x (exonic overlap with reference on the opposite strand)” or “o (generic exonic overlap with a reference transcript)” by the cuffcompare software, were left for the next filter. (3) The remaining transcripts that contained a single exon and were shorter than 200 bp were removed. (4) The remaining transcripts were analyzed by the Coding Potential Calculator (CPC, score < 0) [57], Coding-Non-Coding-Index (CNCI, score < 0) [58], Pfam (E value < 0.001) [59] and k-mer scheme (PLEK, score < 0) [60] software. The transcripts that passed through all these stages were considered to be lncRNAs. The lncRNA expressional levels were reflected as fragments per kilo base per million reads (FPKM).

### 4.6. Identification of circRNA

After the clean reads were aligned to the porcine reference genome, the junctions of the unmapped reads were identified using a back-splice algorithm. Then, the Findcirc software was used to predict the circRNAs [25]. The expressional levels of circRNAs were reflected by mapped back splicing junction reads per million mapped reads (RPM). 

### 4.7. Differentially Expressed RNA Identification and Pathway Analysis

The DESeq software package (http://bioconductor.org/packages/release/bioc/html/DESeq.html) was used to detect differentially expressed mRNAs (DEMs), circRNAs (DECs), and lncRNAs (DELs), with *p* < 0.05, *p_adj_* ≤ 1.00 and | log_2_FoldChange | >1. Gene Ontology (GO) terms and the Kyoto Encyclopedia of Genes and Genomes (KEGG) were performed for DEMs and DECs (using the DECs’ parent genes) using the DAVID tool, and *p* < 0.05 were considered significant [61]. In the GO analysis, the genes associated with muscle growth and fatness were made visible using the Circos software (http://www.circos.ca/software).

### 4.8. Construction of the lncRNA/circRNA-miRNA-mRNA Network

Recently, it was found that mRNAs and lncRNAs/circRNA with the same miRNA response elements (MREs) regulated each other’s expression by competitively binding to miRNAs. To determine the possible interactions between miRNA with mRNA, lncRNA and circRNA, the interactions between the porcine miRNA and the DEMs, DELs, and DECs were analyzed using the miRanda software [62]. The porcine muscular miRNA refers to the published article [63]. The construction of the lncRNAs/circRNA-miRNA-mRNA network was conducted as follows: the miRanda software was used to analyze the relationships between miRNAs and DEMs, DELs, and DECs, and only alignments with energies lower than −20 kcal/mol, with no mismatch in the positions, 2–8 in the 5′ end, remained. The remaining DEMs-miRNA, DELs-miRNA and DECs-miRNA were used to construct the potential regulatory network of lncRNAs/circRNA-miRNA-mRNA and were visualized using the Cytoscape V3.2 software (http://cytoscape.org/) [64]. In the potential lncRNA/circRNA-miRNA-mRNA regulatory networks, mRNAs were the target genes for miRNAs, and the relationship between lncRNAs and mRNAs needed further verification.

### 4.9. Reverse Transcription Quantitative PCR (RT-qPCR)

According to the networks of lncRNA/circRNA-miRNA-mRNA, six nodes around the *MYOD1*, including one miRNA (ssc-miR-296-5p), two lncRNAs (TCON_00034808 and TCON_00016662), and three circRNAs (circRNA_2155, circRNA_1203, and circRNA_6453) were selected, and their expressional trends in the HN and DLY groups were validated by RT-qPCR. The RNA used in the validation was the same as that used in the Illumina sequencing. We used the PrimeScript RT reagent Kit, with the gDNA Eraser (TaKaRa, Dalian, China), to convert the total RNA to cDNA, with random hexamers (for mRNA, lncRNA and circRNA) and stem-loop RT primers (for miRNAs, purchased from RiboBio) [65]. Then, qPCR was performed using the SYBR Green PCR kit (TaKaRa, Dalian, China), according the manufacturer’s instructions. The primers used for the circRNAs were outward-facing, so that they primed divergently and amplified only circularly, but not linear RNA molecules [66]. The glyceraldehyde-3-phosphate dehydrogenase (GAPDH, for mRNA, lncRNA and circRNA) and *U6* (for miRNA) were used as endogenous controls. All primers used in RT-qPCR are shown in Table 1, except for the primers used for ssc-miR-296-5P and *U6*, which were purchased from RiboBio (Guangzhou, China). Each qPCR reaction was performed in a 20 µL reaction mixture, including 20 ng template cDNA, 10 µL 2 × SYBR Premix Ex Taq^™^, and 10 µM forward and reverse primers. The PCR amplification included an initial denaturation step (95 °C for 30 s) and 40 cycles of 5 s at 95 °C and 30 s at 60 °C. Each qPCR experiment was performed in triplicate, and the relative RNA expression values were calculated using the 2^−△△Ct^ method [67]. The data were presented as the fold change in expression.

## 5. Conclusions

In conclusion, we compared the structural and expressional features of mRNAs, lncRNAs and circRNAs in the LD muscle of the Chinese HN and Western DLY pigs. The results showed that ncRNAs were abundant in the porcine LD muscle, and the bioinformatics analyses indicated that ncRNAs were involved in muscular proliferation, differentiation/regeneration and adipogenesis, acting as ceRNAs for vital transcription factors (such as *PPARD*, *MYOD1*, *etc.*) via functional miRNAs (such as ssc-miR-423-5p, ssc-miR-874, *etc.*). This study provided a new insight into the genetic basis of porcine muscle growth and fatness, and further investigations are necessary to validate the ceRNA mechanisms of ncRNAs and mRNAs.

## Figures and Tables

**Table 1:** The primers used for the validation of circRNAs and their expression trends in different groups.

Name	Location	Sequence (5′–3′)	Size (bp)
*MYOD1*	chr2:44482283|44485063	F: AAGTCAACGAGGCCTTCGAG R: GGGGGCCGCTATAATCCATC	279
TCON_00034808	chr9:150794507|150796156	F: CCCCTGTTTACTCACCGTGT R: CCCTCCGTGGCATTTACAGA	94
TCON_00016662	chr17:69254966|69256307	F: TGGAAATCTAATCCTGCGTCTT R: GCGGAAACTCTGCTCCCTAG	105
circRNA_2155	chr13:217685619|217779051	F: ATCTTGGTCGTGCTCCTGG R: CGTGGTTAGTTTCTGCGTTGG	243
circRNA_1203	chr11:85020089|85113533	F: TGTGACATCCCTCTGTACGAC R: GGGAACGGCTTTCTCGT	108
circRNA_6453	chr7:24644089|24722398	F: CGCGGGTACAGTCAGTTTGG R: GGTCACATGTGTCTTTGGAGG	240
*GAPDH*	chr5:66446302|66449510	F: CTGCCCCTTCTGCTGATGC R: TCCACGATGCCGAAGTTGTC	151

## Supplementary Materials

Supplementary Materials can be found at https://www.mdpi.com/1422-0067/20/5/1107/s1.

## Abbreviations

BCLAF1	Apoptosis regulator (BCL2)-associated transcription factor 1
c-JUN	c-Jun Proto-Oncogene
DIAPH3	Diaphanous-related formin-3
DEMs	Differentially expressed mRNAs
Linc-MD1	Muscle-specific long noncoding RNA
ceRNA	Competitive endogenous RNA
circRNA	Circular RNA
CNCICOL3A1	Coding-Non-Coding-IndexCollagen type III alpha 1
CPC	Coding Potential Calculator
DDO	D-aspartate oxidase
DECs	Differentially expressed circRNAs
DELs	Differentially expressed lncRNAs
DLY	Duroc × Landrace × Yorkshire
EBF2	EBF transcription factor 2
FABP4	Fatty acid binding protein 4
FGFR4	Fibroblast growth factor receptor 4
FoxO1	Forkhead box O1
FPKM	Fragments per kilo base per million read
GAPDH	Glyceraldehyde-3-phosphate dehydrogenase
GFPT1	Glutamine-fructose-6-phosphate transaminase 1
GO	Gene Ontology
HN	Huainan
IGF1R	Insulin-like growth factor 1 receptor
IGF2	Insulin-like growth factor 2
IMF	Intramuscular fat
KEGG	Kyoto Encyclopedia of Genes and Genomes
LD	Longissimus dorsi
LPIN1	Lipin 1
LncRNA	Long non-coding RNA
LncRNA ADNCR	Adipocyte differentiation-associated lncRNA
MAML1	Mammalian Mastermind-like 1
MEF2C	Myocyte enhancer factor-2C
miRNA	Micro RNA
MREs	MiRNA response elements
mtDNA	Mitochondrial DNA
MUFA	Monounsaturated fatty acids
MYOD1	Myogenic differentiation 1
NCOA3	Nuclear receptor co-activator 3
ncRNAs	Non-coding RNAs
PDGFRα	Platelet-derived growth factor receptor alpha
PLEK	k-mer scheme
PPARD	Peroxisome proliferator-activated receptor delta
PPARG	Peroxisome proliferator-activated receptor gamma
PRC2	Polycomb repressive complex 2
RIN	RNA integrity number
RPM	Reads per million mapped reads
rRNA	Ribosomal RNA
RT-qPCR	Reverse transcription quantitative PCR
SFA	Saturated fatty acids
TGF-β1	Transforming growth factor beta 1
TOBF1	Transient octamer binding factor 1
VSMCs	Vascular smooth muscle cells
